# Supplementary material for: Analytical Validation of Cxbladder® Detect, Triage, and Monitor: Assays for Detection and Management of Urothelial Carcinoma
Source: Diagnostics (Basel). 2024 Sep 17;14(18):2061. doi: 10.3390/diagnostics14182061 (PMC11431456; doi:10.3390/diagnostics14182061)
Supplement: Supplementary file 1 [file diagnostics-14-02061-s001.zip › diagnostics-3136876-supplementary.pdf]

# Analytical Validation of Cxbladder® Detect, Triage, and Monitor: Assays for Detection and Management of Urothelial Carcinoma

**Justin C. Harvey [1,*], Lisa M. Cambridge [1,2], Charles W. Ellen [1], Megan Colonval [1], Jody A. Hazlett [1], Jordan Newell [3], Xin Zhou [1] and Parry J. Guilford [1,4]**

[1] Pacific Edge Diagnostics NZ, Ltd, 87 St David Street, 9016 Dunedin, New Zealand
[2] Cambridge Quality Consulting, T/A Check Electric Ltd, 204 Coast Road, Warrington, Waikouaiti 9471, New Zealand
[3] Pacific Edge Diagnostics USA, Ltd, 1214 Research Boulevard, Hummelstown, PA, USA
[4] Department of Biochemistry, University of Otago, 710 Cumberland Street, Dunedin 9016, New Zealand
* Correspondence: justin.harvey@pacificedgedx.com; Tel.: +6434795805

**Supplementary Materials**

**Table S1.** Quality control RNA concentrations for extraction control samples.

| Biomarker gene ($log_{10}$ copies/μL) | Expected value (lower limit–upper limit) | | |
|---|---|---|---|
| | HEC | LEC | NEC |
| *CDK1* | 3.39 (2.86–3.92) | 2.06 (1.42–2.70) | 0 (0–1.526[a]) |
| *MDK* | 3.37 (2.8–3.94) | 2.11 (1.43–2.79) | 0 (0–1.526[a]) |
| *IGFBP5* | 3.24 (2.68–3.80) | 2.0 (1.33–2.67) | 0 (0–1.526[a]) |
| *HOXA13* | 3.37 (2.86–3.88) | 2.03 (1.36–2.71) | 0 (0–1.526[a]) |
| *CXCR2* | 3.13 (2.60–3.65) | 1.71 (0.96–2.46) | 0 (0–1.526[a]) |
| IC RNA | 2.75 (2.22–3.28) | 2.59 (2.13–3.05) | 2.52 (2.10–2.95) |

[a]Limit of quantification. *CDK1*, cyclin-dependent kinase 1; *CXCR2*, C-X-C motif chemokine receptor 2; HEC, high-extraction control; *HOXA13*, Homeobox A13; IC, internal control; *IGFBP5*, insulin-like growth factor binding protein 5; LEC, low-extraction control; *MDK*, midkine; NEC, negative-extraction control.

**Table S2.** Quality control parameters for the reference RNA standard curve and calibration RNA sample.

| **Biomarker gene** | **Standard curve slope** | **Calibration RNA concentration, $\log_{10}$ copies/μL** |
|---|---|---|
| | **Expected value (lower; upper limit)** | **Expected value (lower; upper limit)** |
| *CDK1* | −3.27 (−3.57; −2.98) | 5.40 (5.22; 5.59) |
| *MDK* | −3.45 (−3.73; −3.17) | 5.13 (4.91; 5.35) |
| *IGFBP5* | −3.26 (−3.64; −2.88) | 4.66 (4.46; 4.87) |
| *HOXA13* | −3.28 (−3.53; −3.03) | 3.88 (3.64; 4.13) |
| *CXCR2* | −3.23 (−3.53; −2.93) | 2.37 (2.08; 2.66) |
| IC RNA | −3.23 (−3.59; −2.88) | 3.61 (3.43; 3.78) |
| MSE (all biomarkers) | 1 (0; 3.5) | – |
| DoF (all biomarkers) | 6 (4; ∞) | – |

∞, infinity; *CDK1*, cyclin-dependent kinase 1; *CXCR2*, C-X-C motif chemokine receptor 2; DoF, degrees of freedom; *HOXA13*, Homeobox A13; IC, internal control; *IGFBP5*, insulin-like growth factor binding protein 5; *MDK*, midkine; MSE, mean squared standardized error.

**Table S3.** Equations used to determine assay accuracy.

| Parameter | Equation |
|---|---|
| Sensitivity | 100 × [true positives ÷ (true positives + false negatives)] |
| Specificity | 100 × [true negatives ÷ (true negatives + false positives)] |
| PPV | 100 × [true positives ÷ (true positives + false positives)] |
| NPV | 100 × [true negatives ÷ (true negatives + false negatives)] |

NPV, negative predictive value; PPV, positive predictive value.

**Table S4.** Difference in Cxbladder scores of freeze/thaw and non-freeze/thaw reagents compared with expected scores from precision experiment.

| Cxbladder score | RNA standard | | |
|---|---|---|---|
| | **1000 ag** | **120 ag** | **30 ag** |
| Expected score | 0.890 | 0.332 | 0.055 |
| Non-freeze/thaw reagents | 0.911 | 0.394 | 0.112 |
| Freeze/thaw reagents (5 cycles) | 0.898 | 0.326 | 0.080 |
| Non-freeze/thaw vs freeze/thaw difference | 0.013 | 0.068 | 0.032 |

ag, attogram ($10^{-18}$ g)

**Table S5.** Shift in RT-qPCR quantitative cycle values for the five biomarker genes and internal control RNA with three freeze/thaw cycles.

| **Biomarker gene** | **Δ Cq** |
| --- | --- |
| *CDK1* | 0.1562 |
| *MDK* | 0.1743 |
| *IGFBP5* | 0.1855 |
| *HOXA13* | 0.3169 |
| *CXCR2* | 0.1481 |
| IC RNA | 0.1327 |

ΔCq, shift in quantitative cycle value; *CDK1*, cyclin-dependent kinase 1; *CXCR2*, C-X-C motif chemokine receptor 2; *HOXA13*, Homeobox A13; IC, internal control; *IGFBP5*, insulin-like growth factor binding protein 5; *MDK*, midkine; RT-qPCR, quantitative reverse transcription-polymerase chain reaction.

**Table S6.** Tolerable percentages of process-related potentially interfering substances.

| | **Tolerable percentage of inhibitor (%)a** | **Tolerable volume per 64 μL elution (μL)** | **Subjective risk appraisal** |
|---|---|---|---|
| Absolute ethanol | 3.750 | 2.4 | Very low |
| Acetone | 3.750 | 2.4 | Very low |
| Cxbladder stabilizing reagent | 0.750 | 0.5 | Very low |
| MagMAX wash buffer | 1.875 | 1.2 | Very low |
| MagMAX beads | 25.000 | 16.0 | Very low |

[a]Percentage of total RT-qPCR volume. RT-qPCR, reverse transcription-quantitative polymerase chain reaction.

**Table S7.** Intra- and inter-assay variability across high- and low-extraction controls for Cxbladder assays at PEDUSA and PEDNZ.

| | **PEDUSA** | | **PEDNZ** | |
|---|---|---|---|---|
| | **HEC** | **LEC** | **HEC** | **LEC** |
| Intra-assay variability, CV% | | | | |
| *CDK1* | 1.04 | 2.73 | 0.83 | 2.66 |
| *MDK* | 1.73 | 3.28 | 1.44 | 3.70 |
| *IGFBP5* | 1.89 | 3.12 | 1.18 | 3.02 |
| *HOXA13* | 1.56 | 3.03 | 1.11 | 3.31 |
| *CXCR2* | 2.03 | 6.85 | 1.46 | 4.25 |
| IC RNA | 1.14 | 1.35 | 1.24 | 2.33 |
| Inter-assay variability, CV% | | | | |
| *CDK1* | 1.05 | 2.67 | 2.08 | 3.49 |
| *MDK* | 1.70 | 2.47 | 2.78 | 5.26 |
| *IGFBP5* | 0.58 | 1.74 | 2.74 | 4.32 |
| *HOXA13* | 0.89 | 2.68 | 2.38 | 4.02 |
| *CXCR2* | 1.82 | 4.66 | 1.75 | 6.79 |
| IC RNA | 2.02 | 2.44 | 3.66 | 2.60 |
| Total-assay variability, CV% | | | | |
| *CDK1* | 1.47 | 3.82 | 2.24 | 4.38 |
| *MDK* | 2.43 | 4.11 | 3.13 | 6.42 |
| *IGFBP5* | 1.98 | 3.57 | 2.98 | 5.27 |
| *HOXA13* | 1.80 | 4.05 | 2.63 | 5.21 |
| *CXCR2* | 2.73 | 8.29 | 2.27 | 8.01 |
| ICRNA | 2.32 | 2.79 | 3.87 | 3.49 |

CV%, coefficient of variation; HEC, high-extraction control; LEC, low-extraction control; PEDUSA, Pacific Edge Diagnostics, USA; PEDNZ, Pacific Edge Diagnostics, NZ.
